# Autologous platelet lysate local injections for the treatment of refractory lateral epicondylitis

**DOI:** 10.1186/s13018-016-0349-2

**Published:** 2016-01-25

**Authors:** Xun-xiang Tan, Hai-yang Ju, Wei Yan, Hong-jiang Jiang, Jin-ping Su, Hua-jun Dong, Ling-shuang Wang, De-bao Zou

**Affiliations:** Department of Bone and Joint Surgery, Wendeng Orthopaedic Hospital of Shandong Province, Wendeng, Shandong 264400 China; Zhejiang Xingyue Biotechnology Co., Ltd, Hangzhou, 311121 China

**Keywords:** Autologous platelet lysates, Lateral epicondylitis, VAS score, Mayo score

## Abstract

**Background:**

The purpose of this study was to investigate the efficacy and safety of autologous platelet lysate (APL) local injections in reducing pain and improving function in patients with refractory lateral epicondylitis.

**Methods:**

A total of 56 patients with refractory lateral epicondylitis were enrolled in this study. All the patients received three injections in one course of treatment. Subjective assessments include visual analog scale (VAS) pain score and Mayo elbow score before injection (baseline) and at 1, 6, and 12 months after injection.

**Results:**

Significant differences were observed in VAS and Mayo scores at baseline and at 1, 6, and 12 months after injection. Overall, the injections of APL improved local symptoms and all the patients recovered to normal elbow function with 12 months follow-up.

**Conclusions:**

Local injections of APL resulted in favorable clinical outcomes for the treatment of lateral epicondylitis. APL could be clinically effective in the treatment of lateral epicondylitis.

## Background

Lateral epicondylitis (LE), commonly called tennis elbow, is one of the most common enthesopathies in sports medicine. The primary symptoms included persistent pain and tenderness over the lateral epicondyle with the inability in carrying out activities of daily living (ADL). LE affects about 1–3 % of the general population and peaks in the fourth and fifth decades of life [1–3].

A wide variety of therapies have been trialed in the treatment of LE. Initially, treatments for LE include rest, stretching, bracing, formal physical therapy, small needle-knife therapy, anti-inflammatory medication, and activity modification. Cortisone is the next option in patients who do not respond to these treatments. Although the majority of patients improve with conservative management, surgery is needed in 10 % of cases in whom these treatments are ineffective. Besides, these treatments only can improve the symptom; but for the intractable and refractory LE, the best management strategies remain controversial [4].

Most specialists think that the pathology of LE is degenerative, the noninflammatory, chronically degenerative changes in the extensor carpi radialis brevis muscle origins being identified during surgery [5]. The nature of the disease is common extensor tendon injuries aggravated by active and resisted wrist extension and/or repetitive rotation and extension and flexion of the forearm [6].

With the development of molecular biology and regenerative medicine, the use of endogenous growth factors has shown promising results in vitro and in vivo in LE treatment. Numerous studies have examined the effects of growth factors; demonstrating benefits include improved cellular remodeling and decreased healing time [7].

Plasma rich in growth factors has been proved to be safe and effective in treating tendinopathy such as Achilles tendinopathy, patellar tendinopathy, and rotator cuff tears in sports medicine. They have been shown to induce the migration of reparative cells to an injection site, reverse the pathological changes of the enthesiopathy, restore normal structure, relieve symptoms, and reduce the recurrence [8–10].

Autologous platelet lysates (APL) contain a myriad of bioactive growth factors such as vascular endothelial growth factor, insulin-like growth factor, and transforming growth factor; these histo-promotive substances could influence tissue repairing through angiogenesis, cellular chemotaxis, removal of tissue debris, and reconstruction of extracellular matrix [11, 12].

Thus, we discussed the potential roles and advantages of APL and conducted the retrospective study to evaluate the short-term efficacy of local APL injections in reducing pain and improving function in patients with LE. Great clinical improvement with APL therapy is expected.

## Methods

### Patients

This study was approved by the ethics committee of Wendeng Orthopaedic Hospital of Shandong Province. Written informed consents were obtained from all participants.

Between June 2012 and May 2014, 56 patients (21 men and 35 women) who met the eligibility criteria were invited to enter the trial. The average age at the primary presentation of symptoms was 45 years (range, 36–62). The average time from the primary presentation of symptoms to the treatment was 9.7 months (range, 6–18). All the patients were followed up through outpatients after the treatment.

### Eligibility criteria

Patients with severe lateral elbow pain resisting wrist and forearm extension and patients who had persistent pain and tenderness over the lateral epicondyle and affected ADL were involved. Diagnosis of LE was confirmed on an ultrasound. Patients were required to have symptoms for at least 3 months following initial presentation and to have failed conservative treatments. Sixteen patients only received the treatment of massage, acupuncture, physiotherapy, and nonsteroidal anti-inflammatory medication. Thirteen patients only received the treatment of steroid, including steroid unguent and steroid injection. Seventeen patients received all the conservative treatments above. All patients had no significant improvement of symptoms 6 to 14 weeks after the treatments.

Patients were excluded if they met any of the following conditions: patients with cervical spondylosis, any trauma or prior surgical intervention to the elbow, history of inflammatory arthropathy or a tendon tear, rheumatic joint disease, joint limitations due to a previous radial and ulna bone fracture, local osteoporosis, and neurological deficits in the ipsilateral upper limb.

### Preparation of autologous platelet lysates

Approximately 60 ml of peripheral vein blood was withdrawn from each patient into a syringe containing 6 ml of a citrate anticoagulant. After transferring into several centrifuge tubes, the peripheral blood were centrifuged at 1048 r min^−1^ for 25 min at room temperature (Thermo, USA) and then three layers were separated. Platelet-rich plasma (PRP) in the middle layer (about 20 ml) was withdrawn and subpackaged into three vacuum tubes (Fisher, USA). This plasma contains concentrated platelets about three times baseline in an nonactivated form. Then, the tubes were cryopreserved at −80 °C overnight, and then, one of them was resuscitated in a 37 °C water bath kettle for 5 min. After repeatedly freeze-thawed more than twice, the plasma contains a variety of growth factors including platelet-derived growth factor, transforming growth factor-beta, and vascular endothelial growth factor.

The thawed and activated plasma was centrifuged at 3054 r min^−1^ for 6 min (centrifugation radius is 9 cm) to separate the platelet fragmentation in the under layer. Deoxycycline (APP Pharm, USA) of 10 ug ml^−1^ was added into the supernatant with a volume ratio of 1000:1, and then, the APL which contains the cocktail of factors released by the platelets was obtained after filtration. The mean volume of APL injected in our series was 3 ml for each infiltration.

### Injection technique

1. The patient was asked in the sitting position. Confirm the area to be treated by a tiny q-tip, mark the maximum tenderness point, and sterilize with an iodine solution.
2. A 25-G needle was inserted perpendicularly into the periosteum. After withdrawing about 0.5 cm, 3 ml of the buffered APL was injected into the area of abnormality. A single skin portal with multiple penetrations into the tendon by sloping the needle to 60° is recommended so that the liquid disperse fully into the humerus muscular fasciae. The preferred method is to do this procedure under ultra-sonic guidance [13].
3. Tampon the treated area for 3–5 min after injection without moving the involved elbow. The injection was carried out once a week with three injections in one course of treatment.

### Post-injection protocol

Vigorous activities such as strong gripping or lifting should be avoided 1 week post-procedure. The use of nonsteroidal anti-inflammatory medication was prohibited for the first 4 weeks after injection. Icing and elevation are recommended if necessary. After 2 weeks, gradual strengthening and return to activity were initiated.

### Evaluation methods

Patients were evaluated using a visual analog scale (VAS) to assess pain and Mayo score to assess elbow function and patient satisfaction. The VAS score is a measurement instrument to quantify the amount of pain reported by the patient and scores range from 0 (no pain) to 100 (severest pain) with five categories of pain (no pain 0, mild pain 1–30, moderate pain 40–60, severe pain 70–90, and the intolerable pain 100). The Mayo score could reflect elbow function of the patient with full mark of 100 include pain, movement, stability, and ADL (Table 1). The VAS and Mayo scores were recorded prior to the first procedure and at 1, 6, and 12 months follow-up. Complications and patient satisfaction were also recorded.

**Table 1:** Mayo clinic performance index for the elbow

Mayo index	Points
Pain intensity	
No pain	25
Mild occasional	20
Moderate, tolerable	15
Severe to intolerable	0
Functional status	
Returned to regular employment	25
Restricted employment	20
Able to work, but unemployed	15
Unable to work because of pain	0
Range of motion (% of normal side)	
100 %	25
75–100 %	15
50–75 %	10
25–50 %	5
0–25 %	0
Grip strength (% of normal side)	
100 %	25
75–100 %	15
50–75 %	10
25–50 %	5
0–25 %	0

Longitudinal ultrasonography was performed on all patients before injection and at 1 month after APL treatment. Patients were examined in a sitting position with the elbow flexed to 90°, the wrist pronated, and the arm resting on a table [14]. We used a high-quality ultrasound scanner (EUB 900, Hitachi Medical Europe, Zug, Switzerland) with a 14-MHz linear transducer. The transducer was aligned with the long axis of the radius over the common tendon origin. The common tendon origin was examined with color Doppler US in the longitudinal plane by moving the transducer from side to side, locating the part with the most Doppler activity. The color Doppler activity is usually seen in an area limited proximally by the tip of the lateral epicondyle and distally by the humeroradial joint space. The superficial border was the most superficial fibers, and the deep border was the bone [14]. The size of the color Doppler activity area is on behalf of the blood signal strength, and the richer the blood signal, the heavier the degree of inflammation.

## Results

Fifty-six patients met our inclusion criteria. All the patients were followed up through outpatients after the treatment. The overall average length of follow-up was 12 months (range, 9–16).

### VAS pain scores

**Fig. 1:**
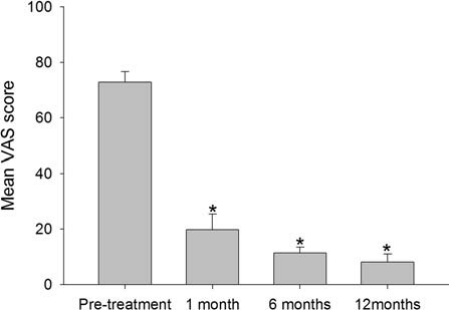
Mean VAS scores of patients at baseline and at 1, 6, and 12 months after injection. *P < 0.05 compared with the baseline

The average VAS pain scores at baseline were 72.8 ± 3.9 for all patients (Table 2), while mean VAS pain scores at 1 month follow-up were 19.8 ± 5.6 and the scores had decreased to 8.2 ± 2.8 at 12 months follow-up (Fig. 1). Particularly, at 1 month follow-up, the average VAS score decreased by nearly 40 points from baseline and there was a significant difference before and after APL treatment (*P* < 0.01). The results showed that the application of APL could reduce pain significantly.

**Table 2:** Mean VAS and Mayo scores at baseline and 1, 6, and 12 months after treatment with APL

	Pre-treatment	1 month follow-up	6 months follow-up	12 months follow-up	*P* value
Mean VAS score	72.8 ± 3.9	19.8 ± 5.6	11.3 ± 2.2	8.2 ± 2.8	<0.01
Mean Mayo score	61.9 ± 3.0	89.6 ± 4.3	92.5 ± 3.3	97.6 ± 2.1	<0.05

Data are presented as mean ± SD

### Mayo elbow scores

**Fig. 2:**
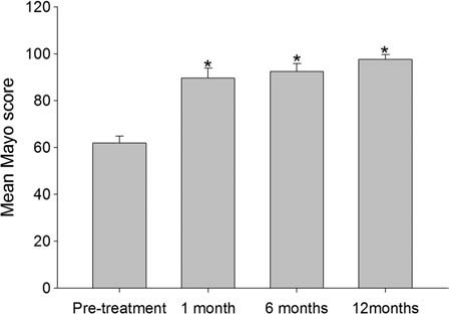
Mean Mayo scores of patients at baseline and at 1, 6, and 12 months after injection. *P < 0.05 compared with the baseline

Significant differences were observed relative to Mayo score (*P* = 0.05) before and 1 month after APL injection. Specifically, at 1 month after APL treatment, the mean Mayo score had increased from 61.9 ± 3.0 to 89.6 ± 4.3 (Fig. 2). Mayo elbow scores had improved 44 % after APL treatment. Furthermore, the mean Mayo scores were 92.5 ± 3.3 at 6 months and 97.6 ± 2.1 at 12 months (Table 2), reflecting good elbow function improvement and efficacy after being treated with APL.

### US evaluation

**Fig. 3:**
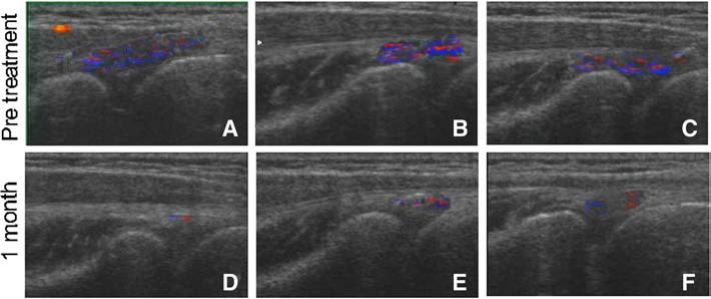
Ultrasonogram graphs of three representative cases before APL injection (**a**–**c**) and 1 month after APL treatment (**d**–**f**). The areas of color Doppler activity were much smaller at 1 month after APL treatment than those before injection in three patients

We presented three cases, and representative ultrasonogram graphs are shown in Fig. 2. The color Doppler activities were very evident before injection (Fig. 3a–c). However, the areas of color Doppler activity were much smaller at 1 month after APL treatment in three patients (Fig. 3d–f). The results meant APL alleviated the inflammation of LE.

### Complications

No adverse events were reported during the trial or at the final follow-up of 12 months. There were no infections, and no patient required any further surgery. At the final follow-up, all the patients had superior elbow function and got good satisfaction.

## Discussion

A tennis elbow patient who received conservative treatment without relief of symptoms for 6 months is difficult to handle, and surgical management such as minimally invasive surgery or arthroscopy surgery is usually needed.

LE is typically attributed to activities that require the repetitive use of soft tissues. Overuse of extensor tendon leads to lower tendon repairing ability, and pathological changes were observed. Histopathology studies in LE reveal degeneration and dysfunction; histological analysis reveals increased vascularity, thinner and disordered collagen fibers, and large interfibrillar mucoid patches and vacuoles. Microscopic evaluation of the tendons does not show any signs of inflammation [15].

Numerous clinical trials have been advocated for treating LE. Rompe extracted data from 10 clinical trials and demonstrated the effectiveness of shock wave therapy (SWT) for lateral elbow tendinopathy under well-defined, restrictive conditions only [16]. Their analysis suggested that there was no consensus for differentiating between low-energy and high-energy shock waves as multiple physical variables are involved. Their research has further identified components that may possibly have an adverse effect on the clinical outcome: enrolment of acute, previously untreated patients; repetitive application of low-energy SWT at monthly intervals; the use of local anesthesia; and follow-up less than 3 months.

Several studies investigated the fundamental role of the platelets in releasing factors able to support and accelerate the healing process. Edwards [17] thinks the underlying pathology of LE is not an inflammatory tendinitis but a degenerative tendinosis; thus, anti-inflammatory drugs should be replaced by a more biological approach for tissue renovation. Autologous blood injections were carried out by them, and 79 % of the patients had significant pain relief after treatment. Mishra and Pavelko [18] performed a cohort study comparing local injection of buffered PRP with bupivacaine in 140 patients with chronic elbow tendinosis. The results showed that 8 weeks after treatment, PRP group noted 60 % improvement versus 16 % improvement in the control group in their VAS scores. At the final follow-up, PRP group reported 93 % reduction in pain compared with before the treatment.

As a form of autologous cytokine therapy, PRP has been investigated to induce the migration of reparative cells to the injured tendon and enhance tendon vascularity and improve the biomechanical properties, but not all types of PRP have the same bioactivity. Traditional PRP contains an increased concentration of white blood cells (WBCs); Anitua thinks that these leukocytes could cause pro-inflammatory effects due to the presence of proteases and acid hydrolyses [19]. Besides, as a type of WBC, neutrophil infiltration has been found to be responsible for the chronic inflammation seen in nonhealing wound; healing of the wound will not occur until the infiltration of neutrophils is reduced. Thus, the use of PRP containing high levels of leukocytes may be questioned [20].

A number of studies have shown the different roles of activated agent in PRP and the healing process chain. Activation of PRP is being discussed in medical literature. After activation, platelets release a burst of proteins from their alpha granules, dense granules, and lysosomes [21]. They contribute the numerous biologically active molecules and provide platelets with their healing properties. Within an hour of activation, 95 % of the granule’s previously manufactured growth factors will be secreted [22].

In this study, we used a freeze-thawing method to activate platelets; after a high-speed centrifugation and supernatant abstraction, APL without leukocytes was obtained. The APL contains pools of bioactive substances and signaling molecules which act as important roles in cell behavior and tissue repair. Crespo et al. [23] confirmed that the addition of platelet lysate to a culture medium could stimulate the proliferation of human mesenchymal stem cell proliferation and chromosomal stability.

The injection of APL into the degenerated humerus is a simple and minimally invasive technique, which helps to promote the restitution of ECM and regeneration of impaired tissue. In this study, we observed highly significant differences relative to VAS and Mayo scores before and 1, 6, and 12 months after local APL injections. Importantly, at the 1 month follow-up, normal elbow functions of all the patients were achieved, reflecting good efficacy. Furthermore, at 12 months post-injection, the patients have slighter pain and recovered to normal bow function.

Our study is associated with several limitations such as retrospective design and the lack of a control group. Sustained efficacy should be further investigated in longitudinal and randomized controlled studies.

## Conclusions

APL local injection therapy is used to initiate response with LE patients who did not respond to conservative treatment for 3 months. Our study findings suggest great clinical improvement for the treatment of LE by APL local injections, and there is no apparent increased risk of complications associated with this procedure. A new, nonsurgical option for the treatment of LE with excellent safety and efficacy was provided through this study; this therapy has the potential to serve as an alternative to surgical intervention, and thus, health care costs were reduced. Limitations of this preliminary study include lack of a control group, lack of objective assessments, small sample size, and the short-term results, and further study is needed. A larger, multicenter, randomized controlled trial is required to further assess the role of APL in refractory LE treatment.
